# Float like a Butterfly: Comparison between Off and On-Ice Torso Kinematics during the Butterfly Stance in Ice Hockey Goalkeepers

**DOI:** 10.3390/s22197320

**Published:** 2022-09-27

**Authors:** Stuart A Evans, Rodrigo Bini, Gregory Davis, James Lee

**Affiliations:** 1 College of Health and Human Science, Charles Darwin University, Darwin, NT 0810, Australia; 2 La Trobe Rural Health School, La Trobe University, Bendigo, VIC 3550, Australia; 3 School of Science, Psychology and Sport, Federation University Australia, Mount Helen, VIC 3842, Australia

**Keywords:** wearables, accelerometer, centre of mass, ice hockey goalkeepers

## Abstract

In ice hockey, the butterfly style/stance is a technique distinguished by the goalkeepers (goalie) dropping to their knees to block attempts to score. Although this goalie style has been around for many years, comparisons between on and off-ice attire has not been undertaken. Therefore, this preliminary study compared differences in torso acceleration and energy expenditure by way of the Metabolic Equivalent of Task (MET) during off-ice and on-ice butterfly stances/saves. Seven participants each performed 8 on-ice butterfly saves/stances whilst wearing full hockey attire followed by 8 off-ice butterfly stances without wearing full hockey attire whilst torso acceleration was collected. The off-ice movement significantly increased vertical torso acceleration (*p* < 0.01, *d* > 0.90) with increased MET, compared to on-ice motion. Despite no significant difference in anteroposterior and mediolateral torso kinematics, vector magnitudes were significantly greater (*p* < 0.01, *d* > 0.90) when the stance was performed off-ice. The increased vertical acceleration observed when goalies performed the movement off-ice could be due to a failure to maintain adequate posture without the support of the external load. The results of this study may help inform off-ice training interventions for ice hockey goalkeeping.

## 1. Introduction

Ice hockey is a high intensity sport that requires versatile biomechanical demands for athletes. Successful performance in ice hockey necessitates intervals of maximal physical strength and power [1,2] and endurance [3], making it a physiologically and kinematically, challenging sport. Physical, neuropsychological, physiological, and anthropometrical differences between ice hockey players, based on their positions—namely forwards (offensive) and defenders (defensive), have been studied over the years by researchers using a wide array of protocols. Nevertheless, most of the prior investigations that occurred have focused on differences between offensive and defensive positions while neglecting goalkeepers. This is largely due to the goalkeeper’s different role and positional demands [4,5,6]. In this regard, the current scientific literature presents limited data in this group of highly specialized ice hockey players.

**Figure 1:**
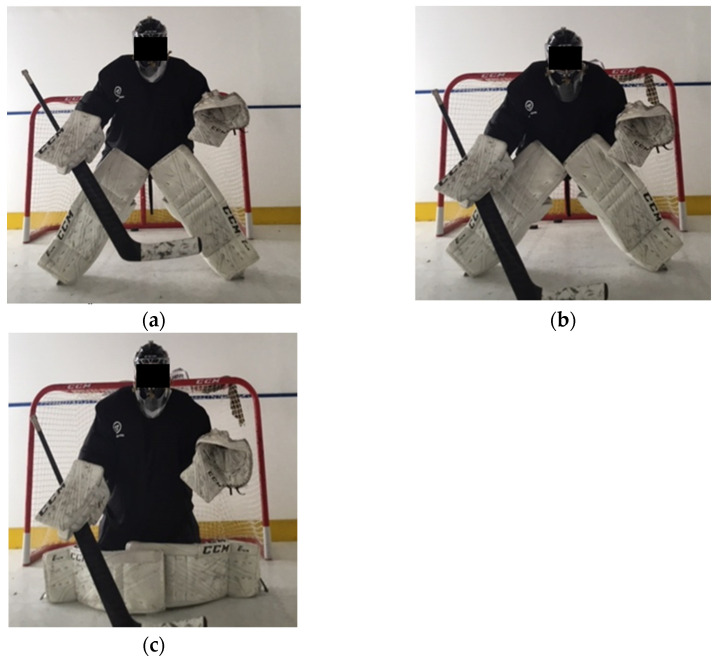
Diagrammatic model of the different components of goalkeeper movements in the butterfly stance performed in the crease. (**a**) Indicates stance positions, (**b**) indicates transition movements, (**c**) indicates final position.

An ice hockey goalkeeper is usually positioned in a designated area in front of the net to cover the goal. The goalkeeper then applies different save techniques in order to prevent the opponent from scoring. Led by Patrick Roy, a then new technique brought the game to its knees by introducing a more effective way of goalkeeping [7]. This technique involves a goalkeeper frequently dropping down to the knees, using extremes of hip range of motion to quickly push from post to post, covering as much of the net as possible [8]. In contemporary ice hockey, the goalkeeper saves most of the shots by dropping down on to the ice into what is known as the butterfly stance (Figure 1).

During a regular National Hockey League (NHL) game, a goalkeeper makes about 45 saves in a full or half butterfly position [9] and drops down in the butterfly position many more times during each game and daily practices. The butterfly technique is unique amongst goalkeepers with this particular style prominent for nearly 20 years [10]. This technique allows the goalkeepers good coverage and readiness to make the save against low angled shots. This is why the butterfly save has become the most used save technique in contemporary ice hockey. While this revolution in style may have a toll on players’ hip and groin health [11], little is known about the role of the torso. Thus, the current scientific literature presents limited data on goalkeeper specific characteristics.

The testing of on-ice and off-ice parameters is fundamental to determine the specific performance of ice hockey players. In preceding studies, off-ice performance (e.g., sprinting and agility tests) were analyzed to determine the relationship to hockey playing [12,13]. Because goalkeepers have a different role to play than defenders and forwards, they have different physical and physiological demands that may need to be trained accordingly [14,15]. Specifically, goalkeepers require technical and mental skills that include quickness, agility, speed, explosiveness, flexibility, fast reaction time, eye-hand coordination, and fast decision making [10]. Consequently, the interchange of on-ice and off-ice strength and conditioning is generally recommended. 

According to the scientific literature, the total game time for ice hockey players, excluding goalkeepers, is approximately 15 to 25 min, depending on the position of the player [3,14]. In contrast, goalkeepers usually play all three periods (3 × 20 min) with occasional overtime. Instead of two- to five-minute breaks between shifts, goalkeepers get roughly 15 to 18 min of rest between each period and some smaller rest periods when the game shifts to the other side of the ice [16]. As a consequence, greater physical demands are placed on the goalkeeper of the short-handed team who has to move laterally and vertically more often [10]. The differences in lateral and vertical movement also suggest that greater kinematics demands are placed on goalkeepers in these anatomical planes. However, current understanding of both physiological and kinematical demands in ice hockey goalkeepers is mainly based on collegiate athletes and elite-level players, and research on semi-professional players is sparse. Therefore, it is important to evaluate these movements using devices that are able to detect such variables. 

Physical activity refers to bodily movements produced by skeletal muscles that expends energy [17]. The amount and the intensity of physical activity when goalkeepers perform the butterfly stance can be assessed by energy expenditure. The Metabolic Equivalents of Task (MET) are multiplies of the resting metabolism that reflects metabolic rate during exercise. This energy cost can be expressed by the MET that mirrors the metabolic rate [18]. The standard MET is defined as 3.5 mL/kg^1^ min^1^. Despite the prevalence of the MET, use of the MET metric in ice hockey is minimal. Such information could be useful for coaches when comparing off-ice training to on-ice requirements given the evidence that off-ice training may have a motor transfer effect to on-ice agility [10]. 

Existing research has primarily examined hip kinematics during the butterfly save [19] in an attempt to relate selected on-ice and off-ice strength and anthropometric determinants to skating speed [11]. Despite this, the goal of lower-limb movement is the forward translation of the body system, which can be mechanically represented by its centre of mass (CoM). In this way, the torso and CoM transfer and control force and motion in an integrated kinetic chain. 

A common method used to facilitate a post-activation potentiation response is performing sport specific movement patterns with added resistance that provides additional resistance to movement. An ice hockey goalkeeper can expect their on-ice attire to weigh approximately between 14–20 kg (dry weight) [20]. In this instance, the attire provides additional resistance to movement. This resistance may alter kinematical parameters as well as impact energy expenditure by way of the MET. Practically, by imitating on-ice movements in an off-ice environment, desired adaptations could be obtained that allow players to optimize technical skills. To more closely replicate on-ice patterns, off-ice movements can be performed with a resistive load since adding resistance would reduce the rate of acceleration and increase ground contact time [21] which may alter the MET. Ice hockey, though, has no true off-ice equivalent, even though this element is utilized during training. It is therefore critical to have appropriate biomechanics in order to replicate on-ice demands. However, the role that ice hockey attire has on torso kinematics and energy expenditure is unclear.

In recent years, new technologies such as inertial sensors based on Micro Electro Mechanical Systems (MEMS) have been developed, which have low production costs, are small in size and have the ability to measure kinematics over large periods of time [22,23,24]. Moreover, studies have investigated the usefulness of these devices in different areas including lower limb biomechanics [25], movement sport specific [26], and sport performance [23]. Despite the exhaustive body of literature on the demands of ice hockey [1,3,9,12,13] less is known about the relationship between functional performance testing protocols (on-ice and off-ice). Consequently, the influence of ice hockey goalkeeper equipment on torso kinematics during the butterfly movement remains inconclusive. The present study aimed to compare differences in torso acceleration and the MET during the butterfly stance with and without attire in off-ice and on-ice environments using a triaxial accelerometer. We hypothesized that significant differences in on-ice and off-ice performance in vertical and anteroposterior magnitudes of acceleration would be present, as well as a low relationship between the MET in on-ice and off-ice environments.

## 2. Design and Participants

Seven (*n* = 7) semi-professional male ice hockey players participated in this preliminary study (age: 36 ± 10 years: body mass without equipment: 87 ± 13.4 kg; body mass with equipment: 101.8 ± 13.4 kg; height: 182.6 ± 5.8 cm; weekly training volume: 8 ± 2.6 h) having been approved by the University Research Ethics Committee (HREC H21114). All of the ice hockey goalkeepers included in the study were training at least once per week for a duration of two hours or more that included on and off-ice activity. Additionally, all of the goalkeepers were playing competitively (defined as an on-ice game against opposition whereby points are gained for the winning team) at least once per week. The participants had at least two years of both on and off ice training including butterfly technique training. Goalkeepers were excluded from the study if they had any injury (acute or chronic) or illness at the time of the test that prevented them from exerting maximum effort. Only players (goalkeepers) over the age of 18 were asked to participate in this study. Before participation in the study, written informed consent was obtained from all of the goalkeepers. 

The participants were informed that they could withdraw from the study at any time without penalty. All of the goalkeepers were informed of the experimental procedures, risks, and benefits, after which they signed an informed consent form before participating in the study. Furthermore, the goalkeepers were recruited by word of mouth and were registered with the Australian Ice Hockey League (AIHL) or Ice Hockey Victoria (IHV). None of the goalkeepers reported any current or ongoing neuromuscular diseases or musculoskeletal injuries specific to the ankle, knee, or hip joints, and no participant reported taking any dietary or performance supplements that might be expected to affect performance during the study. All the participants were fully accustomed with the procedures used in this research and were informed that they could withdraw from the study at any time without penalty.

### 2.1. Materials and Methods

This was a randomised and crossover study that was performed in an on-ice and off-ice environment. The study design consisted of two parts that included participants randomly drawn into either an on-ice or an off-ice group. Therefore, four of the goalkeepers performed the butterfly stance on-ice while the remaining three goalkeepers performed the stance off-ice. Once completed, the participants changed groups in order to complete the protocol in either an on-ice or an off-ice environment.

**Figure 2:**
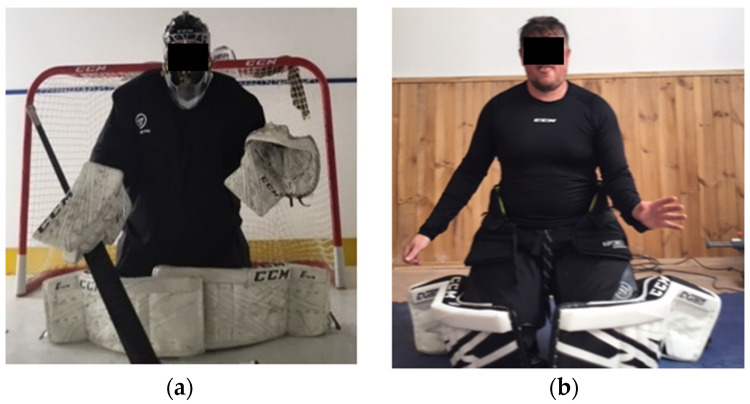
Butterfly stance performed on synthetic ice with full hockey attire (**a**); Butterfly stance performed off the ice (**b**).

For the on-ice component of the study, the goalkeepers were required to complete 8 on-ice deep blocking butterfly stances while wearing characteristic on-ice goalkeeping attire. On-ice hockey apparel consisted of helmet, shoulder pads, padded gloves, padded shorts, shin guards, elbow pads, chest protector, athletic cup groin protector and hockey skates. After an acoustic countdown (“3, 2, 1, go”) participants performed the butterfly technique. For the off-ice component of the study, the goalkeepers were once more required to complete 8 deep blocking butterfly stances while wearing part hockey attire that included shin guards and padded shorts. Thus, data was obtained on-ice (with attire) and off-ice (without full attire) (Figure 2). A duration of approximately 15 min passive recovery separated the on-ice and off-ice tests in order to allow participants to remove the necessary on-ice attire. All participants were familiarised with the on-ice and off-ice butterfly technique protocol before the experiment. Prior to testing, participants completed a self-selected 10-min warm-up that included a combination of on and off-ice butterfly stances, skating back and forth on the synthetic ice, and dynamic stretches. The participants performed three off and on-ice stances as part of the familiarisation process. 

Aerobic metabolism is required for goalkeepers to recover between short and high intensity periods. This is to maintain an alert and ready position for long periods of time, and to make numerous saves during short intense duration [2]. This short intense duration was represented in the current as each butterfly stance was separated by 5 s of passive recovery to replicate the demands of the sport. 

The off-ice tests were performed in a modified indoor setting complete with gymnasium landing mats. The modified gymnasium was 5 × 4 m. The use of landing mats was necessary in order to limit potential injuries during the off-ice tests as participants would have been required to land directly onto solid flooring. To further reduce the risk of injury off the ice, the participants retained lower shin guards and padded shorts whilst performing the butterfly stance to enable safe landing. All participants performed the off-ice tests without footwear. 

The synthetic rink was 6 × 4 m and was made from ultra-high molecular weight polyethylene (UHMWP) sheets (Plastic Warehouse, Hillsdale, NSW, Australia). Each sheet was 13 mm in height (thickness) and assembled into interlocking pieces. The testing procedure used a standardised sequence in that the day started by measuring anthropometric (e.g., body mass) and personal data (age, training experience). All of the participants were measured using traditional digital scales (Eufy Smart Scale P2, Directed Electronics, Australia) with and without hockey attire in order to obtain the weighted mean. A Sportline 240 Econosport stopwatch (New York, NY, USA) was used to monitor time which was manually recorded by the principal author. To limit any outside factors that could potentially change the motion of the participant, data was collected in one day with both tests conducted from 0900–1200. The tests were performed during ambient conditions (19–21 °C, 60–65% relative humidity). The participants were cooled with two vertical standing pedestal fans. 

### 2.2. Instrumentation 

Advances in small and precise accelerometers provide real-time detection of motion [17]. Thus, activity was measured using the ActiGraph GT9X + accelerometer (ActiGraph, LLC, Pensacola, FL, USA). This small, lightweight device (46 × 33 × 15 mm, 19 g) measures acceleration during movement across the vertical (x, upward-downward), anteroposterior (y, forward-backward) and mediolateral (z, side to side) axis. 

Although primarily a physics term, the centre of mass (CoM, also denoted as the balance point) is a theoretical point and an indicator of a body’s position relative to the centralized point of mass. Although the relative position of the CoM will depend on the gender and the location of the limbs, when in the anatomical position the CoM and centre of gravity lies approximately anterior to the second sacral vertebra [24,27]. Specifically, the spinous processes, de-fined as the lumbar vertebrae position 5 (L5) and sacrum vertebrae position (S1), is the basis for this location in that it is the unique and closed external point to the whole-body CoM and point of distribution of the weighted position vectors that sum to zero.

**Figure 3:**
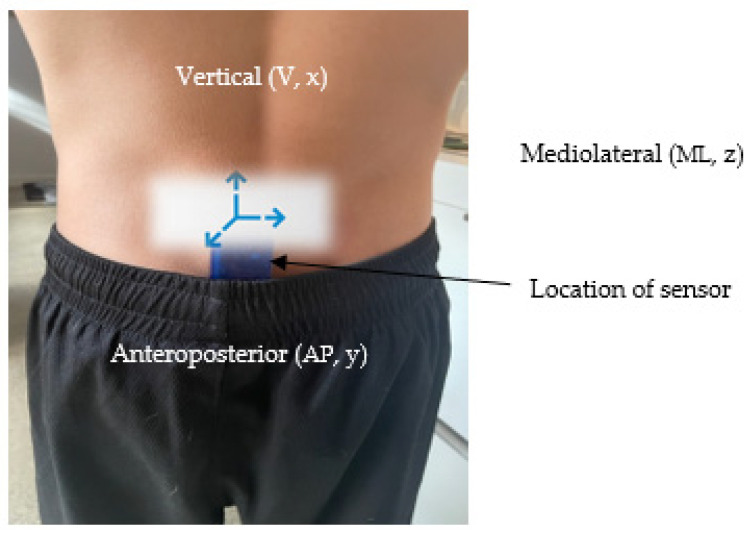
Location of the ActiGraph wearable sensor and associated accelerometric axis on an ice hockey goalkeeper.

The quantification of torso motion can be inferred by the relative amount that the CoM accelerates. This acceleration is made by data output that captures acceleration magnitude in three orthogonal axes where vertical (longitudinal, x, upward-downward), mediolateral (z, side to side) and anteroposterior (y, forward-backward) are aligned with anatomical axis and planes of motion. As acceleration is the derivative of velocity and velocity is the derivative of position with respect to time, trunk acceleration can be presented as meters per second per second, or m/s^2^. The device was secured to the participants by double sided tape between the L5 and S1 spinous process [28,29] [Figure 3].

The ActiGraph device was controlled wirelessly from the principal author via a typical Hewlett Packard PC using the ActiLife software program (Version 6.13.4, ActiGraph, LLC). Only the local acceleration components of the participants were analysed. For repeatability, each participant was assigned a device which was subsequently used for both tests. For each test, the mean torso activity counts and raw acceleration magnitude, the latter expressed in m/s^2^, was determined over the 8 butterfly stances. Thus, the numeric of counts and raw acceleration data are presented. The ActiGraph accelerometer records activity counts in a given time interval, for example, half-minute or minute intervals. Thus, observations are expressed as activity counts per 60 s. Counts are a consequence of summating post-filtered accelerometer values (raw data at 100 Hz) into epoch “chunks.” The value of the counts will vary based on the frequency and intensity of the raw acceleration relative to the goalkeeper’s movement.

Triaxial acceleration data collected from the ActiGraph was converted to activity counts through a process called actigraphy, which results in an interpretable and objective measure of physical activity [30]. The accelerometer output in counts per minute for each butterfly activity was determined by first multiplying the count per second by sixty and then averaging the counts per minute to obtain one count/min^1^ value for the on-ice and off-ice butterfly stances. This method for establishing counts/min^1^ ensured the activity monitor and metabolic measurements were synchronized for each second of data collection. 

For comparison with the Compendium of Physical Activities [31], the METs were summarized by calculating the average MET score from each of the vertical, anteroposterior and the mediolateral axis. The MET value for each was defined as a resting metabolic rate (RMR), assumed at 3.5 mL/kg/min^1^ (corresponding to 1 MET). The MET results are presented for the on and off-ice butterfly stances with direct comparisons with the Compendium code that was used and the corresponding MET value.

Concerning the magnitude of triaxial acceleration, this was a result of summing post-filtered accelerometer values (raw data at 100 Hz) into epoch “chunks” via an ActiLife GT3X file and an AGD file. From here, raw data was converted into a CSV file and manually filtered into epochs based on the frequency and intensity of raw acceleration. The vector magnitude incorporated the vertical, anteroposterior and lateral axes and refers to the length and size of the vector. Thus, vector magnitude was calculated where VM = √ x^2^ + y^2^ + z^2^. Analysis was performed in the time domain with the mean of each epoch analysed. Prior to both tests, the sensor was initialized according to manufacturer instructions. 

## 3. Statistical Analysis

The normality of appropriate data sets was confirmed by the Shapiro–Wilks test using the Analyse-it statistical package (Leeds, UK, version 4.92). The assumptions of normality, linearity, and homogeneity were satisfied. Means and standard deviation (±SD) were analysed for the local acceleration components. Despite the small sample size of participants, parametric tests were deemed appropriate given the assumption of a normal distribution. In contrast, most nonparametric tests use some way of ranking the measurements of the distribution [32], which was not suitable in the current study.

The student’s *t*-test was used to assess differences between on-ice (Test 1) and off-ice (Test 2) with a modified Cohen’s *d* calculated to assess effect sizes and interpreted according to established guidelines where 0.1–0.3 (small), greater than 0.3–0.5 (moderate), greater than 0.5–0.7 (large), greater than 0.7–0.9 (very large) and greater than 0.9 (extremely large) [33]. A paired Student’s *t*-test was used to compare temporal raw magnitudes of acceleration with the MET values. Regression analysis was performed to analyse the relationship between accelerometer counts and the magnitude of triaxial torso temporal accelerations with the MET. The rationale for this approach was to describe and estimate the association between the magnitude of temporal torso accelerations to the MET in both on and off-ice environments. Therefore, a coefficient of determination (*r*^2^) was performed along with two predictive equations that represented on and off-ice environments. Post-hoc processing was completed using raw accelerometry data to assess individual differences in the magnitude of torso acceleration between off-ice and on-ice butterfly stances. A range of ActiGraph counts were considered as cut-points for defining intensity levels. The method of selecting an optimal cut-point was based on two criteria: (1) correct identification of the target or higher intensity activities (i.e., sensitivity); and (2) correct exclusion of lower intensity activities (i.e., specificity). In both tests, means were compared using a priori of 0.05.

## 4. Results

**Figure 4:**
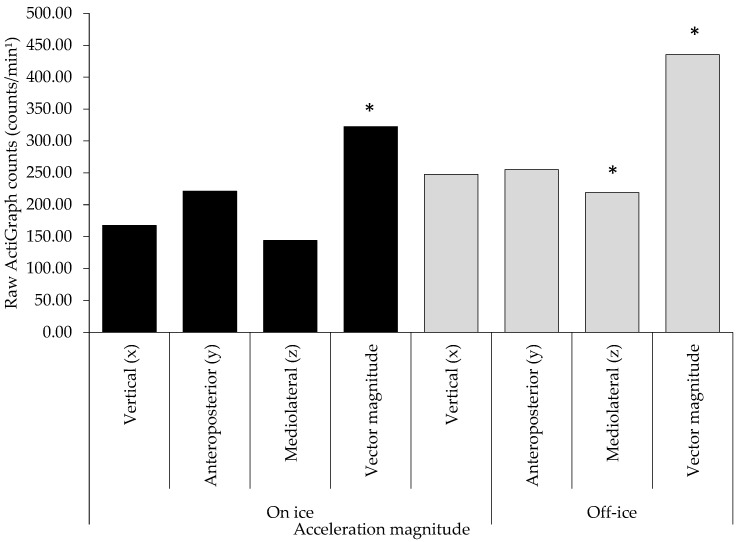
Raw mean ActiGraph counts and magnitudes of triaxial acceleration data in m/s^2^ in 8 on and off-ice butterfly stances. Significant at * *p* < 0.05.

Figure 4 displays the mean raw magnitudes of torso acceleration in the butterfly stance in off-ice and on-ice environments. The increase in the off-ice vector was largely due to the combined effect of greater magnitudes of vertical and anteroposterior torso motion during the butterfly save.

**Figure 5:**
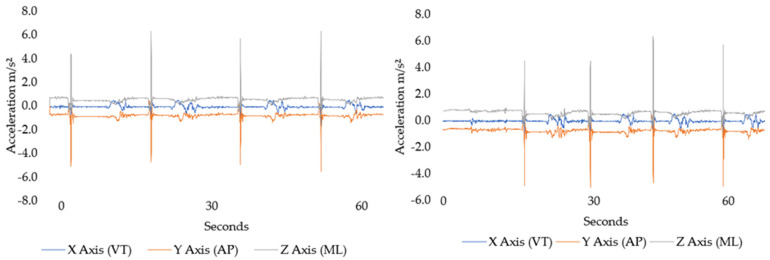
Representative of temporal magnitudes of acceleration pertaining to timeseries data for torso acceleration obtained for one participant during butterfly stance performed on and off-ice for 60 s. Plot shows 6–7 repetitions including passive recovery. Where vertical (x), anteroposterior (y) and mediolateral (z).

Concerning the magnitude of torso acceleration, post-hoc processing revelated that inter individual differences were greater when the butterfly stance was performed off-ice as evident by the wider dispersion of ±SD. Significant differences and extremely large effects were detected in the vertical and mediolateral channels (Figure 5).

Results in the paired *t*-tests (Table 1) indicated significant differences in vertical temporal magnitudes of acceleration in the off-ice butterfly stance. The mean anteroposterior and mediolateral magnitudes of acceleration, although non-significant, resulted in larger effects when the save was performed off-ice. Taken together, the vector magnitude was significantly greater when the stance was performed off-ice. 

The measured MET and the Compendium standard MET are presented in Table 2. For both on and off-ice butterfly stances, the measured MET was not significantly different than the Compendium value in two acceleration axes. This was despite significant differences in the mediolateral axis (*p* < 0.001). Overall, the MET for the vector magnitudes for the on-ice and off-ice butterfly stances did not significantly differ to the Compendium values.

The combination of temporal magnitudes of torso acceleration for the on-ice butterfly technique resulted in a combined intensity value. In this case, the magnitude of acceleration was summed together. The *r*^2^ value for a line of best fit was 0.92 (*f* = 11.97, *p* = 0.179). The best fit line produced the following equation:

On-ice MET mL/kg/min^1^ = 12.57 mL/kg/min^1^ + 0.7774 m/s^2^ (1)

Equally, the grouping of temporal magnitudes of torso acceleration for the off-ice butterfly technique was summated. The *r*^2^ value for a line of best fit was 0.49 (*f* = 0.96, *p* < 0.5). In this instance the line of best fit produced the following equation:

Off-ice MET mL/kg/min^1^ = 14.07 mL/kg/min^1^ + 0.569 m/s^2^ (2)

## 5. Discussion

The present study aimed to compare differences in torso acceleration and the MET during the butterfly stance with and without attire in off and on-ice environments using a triaxial accelerometer. We hypothesized that significant differences would occur in on-ice and off-ice performance in vertical and anteroposterior magnitudes of torso acceleration, as well as a low relationship between the MET in on-ice and off-ice environments. Our study used a randomized and crossover design methodology to understand the differences in longitudinal, mediolateral, and anteroposterior torso accelerations and the associated levels of energy expenditure. Moreover, data capture occurred in-the-field using unobtrusive sensor technology. 

### 5.1. Acceleration Magnitudes of the Torso

The impact of the butterfly stance was assessed through off and on-ice measures that involved performance of a characteristic butterfly stance with and without typical full goalkeeping attire that is worn during on-ice practice and competition. The results from the current study demonstrated that the magnitude of vertical acceleration of the torso was significantly greater when the goalkeepers performed the butterfly save off-ice and without full goalkeeping attire (*p* = 0.0097, *d* > 1). This partially confirms our hypothesis that significant differences would occur in vertical acceleration of the torso. 

In contrast, no significant differences in anteroposterior and mediolateral acceleration were observed between off-ice and on-ice conditions. This is not necessarily surprising since it is clearly known that an on-ice assessment is more specific for ice hockey players [34,35]. In this instance, the results from the present study are in accordance with others given the differences in player movement between on and off-ice environments. Besides that, little research has attempted to shed light on the magnitude of torso acceleration in goalkeepers in a sport that requires significant torso stability and control. 

The butterfly stance is repeatedly performed by goalkeepers during practice and competition. Notably, the butterfly stance provides goalkeepers better coverage of the net due to the widespread movement of the legs. During a butterfly save, the goalkeeper drops down to their knees and flairs their lower leg by maximally internally rotating the hip joint [36]. A greater magnitude of vertical acceleration in the off-ice butterfly stance was probably due to an inefficiency to maintain adequate posture without the support of the external load (e.g., upper body attire). Although the anatomical motion of the butterfly stance is multifaceted and complex, accounting for total load on a goalkeeper’s torso may add potential restrictions in specific actions when off and on-ice. 

The butterfly stance requires goalkeepers to move into a position that requires internal rotation of the femur, external rotation of the tibia, and flexion of the knees. Medial rotation of a limb turns toward the long axis of the trunk [37]. However, good core muscular endurance and strength are needed during abrupt changes in direction for goalkeepers to make saves, and to pass and clear the puck with their stick [38]. While there were no significant differences in anteroposterior and mediolateral acceleration magnitudes of the torso between off and on-ice butterfly the stances, extremely large effects were observed alongside an increase in vector magnitude in the off-ice stance. (Figure 5, Table 2). This may suggest a lack of torso, or core, stability when the stance was performed off the ice. 

A strong core stability allows goalkeepers to keep good balance and protect against contact [38]. Additionally, a high level of abdominal endurance may reduce the risk of injury and lower back pain [14,39]. Therefore, abdominal stability assessment is a pertinent tool to use with goalkeepers as abdominal stability can impact velocity of vertical movement [40]. Despite our result, it is not possible to conclude if the large effects in anteroposterior and mediolateral torso acceleration would influence a goalkeeper’s ability to enact rotational forces to save powerful shots in a replicative competitive environment. 

A goalkeeper’s stance involves unique physical and kinematical demands, such as a high recruitment of hip adductor, flexor, and rotator muscles which are crucial in lateral movement push-offs and good core muscular endurance and strength [41,42]. Additionally, as goalkeepers push off from the ground, the CoM displaces orthogonal to the longitudinal axis of the skate blade [11]. In this regard, when the butterfly technique is performed off-ice and without the high cut hockey boot that is commonly worn when on the ice, the ankle is likely to increase range of motion to accommodate movement. In contrast, when performed on-ice the boot provides stability [11]. The curved blade essentially aids weight transfer from the front to back of the skate, permitting limited movement. This could explain the differences that were seen in the magnitude of acceleration in the anteroposterior and mediolateral off-ice stances (Table 1 and Table 2). Also, the significance of the greater vector magnitude observed off the ice combined with greater activity counts, the latter which is suggestive of increased energy expenditure, may mean that different training methods are needed. Future research could involve the use of sensors to detect torso movements in varied training environments to better understand the limitations of ice hockey attire. In perspective, significant uses of sensors may include strength and conditioning strategies in off and on-ice training modalities. 

### 5.2. Metabolic Equivalents of Task 

The results of the current study demonstrated that the MET was greater when the butterfly stance was performed off-ice. In particular and relative to the Compendium code, the activity for competitive ice hockey has a MET of 10 (code 15362). Despite our MET values being marginally higher compared to the Compendium, overall results remain consistent with others. For instance, our results are consistent with Bassett et al. [43] who found that 15 of 25 measured METs (60%) were significantly different than the Compendium value in a smaller, more homogenous sample. One conceivable explanation for the higher MET values seen in the current study is that the butterfly stance activity can be classified as being performed at a relatively moderate intensity, or not performed under a steady-state condition. Under steady state conditions, at powers below the critical power [44], oxygen uptake ($\dot{V}$O_2_) attains a steady level which can be maintained for a long period of time. In contrast, anaerobic power and capacity are two factors that are well known to be of critical importance in hockey performance [45].

The goalkeeper’s actions are characterized by fast, repetitive, and explosive movements of short duration with periods of lower intensity actions or rest [46]. A potential explanation for the slight variation seen in the MET is that it would be unrealistic to expect all individuals to perform a self-paced yet moderate intensity activity such as the butterfly stance at a constant work rate. Therefore, some MET differences would be expected. However, accurate assessment of inferred energy expenditure, along with acceleration magnitudes of the torso, may help to reveal important components in developing appropriate energy efficiency strategies when training. 

Abdominal endurance is fundamental because it allows for good stability in order for players to perform fast velocity actions [14]. In our study, although greater vertical, anteroposterior, mediolateral and METs were seen when the butterfly stance was performed off-ice (Table 1 and Table 2), inadequate hip flexion or torso strength could viably cause greater acceleration and therefore METs despite the goalkeepers not wearing full hockey attire off the ice. Therefore, a goalkeeper must be physically fit and have adequate torso strength to endure physical activity demands such as the butterfly stance. 

### 5.3. Anthropometry

Our preliminary study included a small sample size (*n* = 7) of semi-professional goalkeepers. Of these goalkeepers, a wide dispersion of age ranges was seen (36 ± 10 years), suggesting variable aerobic, anaerobic, and kinematical differences between the participants. Normally, professional male goalkeepers are significantly older than amateurs, which can be explained in that biological maturity is a predictor utilized during hockey camp selections to determine which players will access higher playing levels [47]. Furthermore, professional male hockey goalkeepers are significantly heavier than their semi-professional and amateur counterparts, which can be explained by the greater amount of time professionals spend on strength and aerobic exercise training [48]. While professional goalkeepers were not included in the current study, this does raise questions regarding the possible influence of age differences that were seen between the seven participants. As a considerable amount of information can be derived by the instantaneous data provided by sensors, the correct interpretation of age-related differences may require additional research. 

The physical fitness of a professional goalkeeper has changed over the years. Off-ice fitness testing is commonly used to predict the physiological abilities of ice-hockey players. For example, there is a notable association between certain off-ice tests (e.g., anaerobic capacity) to on-ice skating acceleration [49]. Whilst sprinting against a resistance has been shown to increase both trunk flexion and stride frequency [21], little research has examined modern goalkeeping techniques. This is despite modern techniques being characterized by more kneeling body postures and therefore increased usage of the butterfly save technique. In this sense, kinematical investigations are essential to comprehend the impact of resisted and unresisted loads during specific movements in both environments. For goalkeepers, the implication is that replicating on-ice movements when off-ice should encompass typical on-ice attire. As verified by Pollitt [50], moving and performing athletic tasks that replicate competition is integral. Given the highly specialised movements, this information may be of practical benefit for coaches to monitor postural stability.

## 6. Limitations and Recommendations for Future Research

Given the small number of participants in this study, the results cannot be generalised or inferred to a wider population. Another limitation is that the participants consisted of semi-professional ice hockey goalkeepers and not elite nor professional players. This might potentially lead to biased comparisons because results from this study are not specifically comparable to goalkeepers from the NHL. However, in the current literature comparisons between on-ice and off-ice kinematical capacity assessments in goalkeepers are lacking. 

Goalkeepers were required to perform off-ice stances in a modified gymnasium setting. This included the use of landing mats to limit potential injuries as participants would have been required to land directly onto solid flooring. Although the landing mat may have provided cushioning when goalkeepers dropped to their knees during the save, this is representative of a typical training situation. Therefore, future research should apply specific criterion for defining the experimental protocol in order to correctly categorise the ability elite, semi-professional, recreational and junior goalkeepers. Testing protocols should also reflect the demands of on-ice competition experienced by the experimental population. Additionally, a continued focus on current strength and conditioning off-ice data when training with and without the addition of equipment will provide a more in-depth understanding of the ever-changing demands of performing the butterfly stance that will in-turn shape the direction of future research. For example, training off-ice with different weighted loads could assist in retainment of movement that matches on-ice energy expenditure, as measured by activity counts. In summary, the results gained from this study could serve as benchmark values for strength and conditioning specialists when they work with goalkeepers of different performance levels. 

## 7. Practical Applications

In summary, the present study explored the effects of a goalkeeper’s ice hockey attire related to kinematical aspects of the butterfly technique and the relative difference between activity counts and the MET. The off-ice butterfly stance resulted in greater vector magnitudes predominately due to larger extents of vertical acceleration. Coaches should be encouraged to teach goalkeepers’ how to perform the butterfly technique prior to on-ice training. Drawing on the results of this study, strategic interventions could aim at improving postural stability through development of refined torso and CoM movement control. The increased vertical acceleration observed when the goalkeeper’s performed the movement off-ice could be due to a failure to maintain adequate posture without the support of the external load. The results of this study may help inform off-ice training interventions for ice hockey goalkeeping. 

## 8. Conclusions

The results from this preliminary study found that the off-ice butterfly stance resulted in greater vector magnitudes predominately due to larger extents of vertical acceleration. This was reflected in the differences between the MET given that a greater MET occurred off-ice compared to on-ice. This was observed despite participants not wearing upper body attire during the off-ice movement. Determining the relationship between the MET, the magnitude of torso acceleration and the on-ice and off-ice test, it may be possible to establish a range of kinematic and physiological responses that are desirable to determine the physical performance of ice hockey goalkeepers. However, caution is needed when extending our results to elite ice hockey goalkeepers. 

Continued research should aim at refining and developing new accelerometry procedures and field-based testing methods. This will enable practitioners to identify goalkeepers susceptible to increased and undesired METs and greater magnitude of torso acceleration in order to improve specific training strategies. 

## Figures and Tables

**Table 1:** Mean maximum acceleration in m/s^2^ for total of 8 butterfly stances performed on-ice and off-ice in three axes inclusive of vector magnitude. * Significant at *p* < 0.05.

Axis	On-Ice Butterfly Stance	Off-Ice Butterfly Stance	*t*	p *	Cohen’s *d*
	Acceleration magnitude (m/s^2^)	Acceleration magnitude (m/s^2^)			
Vertical (x)	−4.74 ± 0.2	−7.61 ± 0.9	1.24	<0.001	>1 (extremely large)
Anteroposterior (y)	−0.43 ± 0.3	0.10 ± 0.5	0.18	<0.148	0.3 (moderate)
Mediolateral (z)	3.23 ± 0.2	5.19 ± 0.7	−0.10	<0.093	>1 (extremely large)
Vector magnitude	8.13 ± 0.1	14.44 ± 1.3	2.12	<0.001	>1 (extremely large)

**Table 2:** Measured MET for on-ice and off-ice activity compared to the nearest and most appropriate MET from the Compendium of Physical Activities. Where the MET value represents 3.5 mL/kg/min^1^ (corresponding to 1 MET). * Significant at *p* < 0.05.

Axis	Off-Ice MET (mL/kg/min^1^) Mean	On-Ice MET (mL/kg/min^1^) Mean	*t*	*p*	Nearest Compendium (MET mL/kg/min^1^) Heading & Description
Vertical (x)	9.36 ± 0.3	10.15 ± 0.8	2.93	0.099	MET 10.0: Conditioning exercise—Calisthenics (e.g., pushups, sit ups, pull-ups, jumping jacks), vigorous effort.
Anteroposterior (y)	10.01 ± 0.4	11.98 ± 0.5	3.46	0.146	MET 10.0: hockey, ice, competitive
Mediolateral (z)	12.2 ± 0.1	13.3 ± 0.9	16.84	<0.001 *	MET 10.0: hockey, ice, competitive
Vector Magnitude	13.93 ± 5.2	14.32 ± 3.2	5.85	0.179	MET 14.0: rollerblading, in-line skating, 24.0 km/h (15.0 mph), maximal effort

## Data Availability

The data presented in this study are possibly available on request. This will be subject to institutional ethical approval for release by the researchers.
